# BrightFocus Alzheimer’s Fast Track 2019

**DOI:** 10.1186/s13024-019-0348-y

**Published:** 2019-12-20

**Authors:** Keith W. Whitaker, Frank M. LaFerla, Harry W. M. Steinbusch, Cynthia A. Lemere, Diane E. Bovenkamp

**Affiliations:** 1 BrightFocus Foundation, 22512 Gateway Center Dr, Clarksburg, MD 20871 USA; 2 University of California, Irvine, USA; 3 Maastricht University, Maastricht, The Netherlands; 4 Brigham and Women’s Hospital, Harvard Medical School, Boston, USA

**Keywords:** Dementia, Alzheimer’s disease, Neurodegeneration, Workshop

## Abstract

The 3 day workshop “Alzheimer’s Fast Track” is a unique opportunity for graduate students, postdoctoral fellows, or other early-career scientists, focused on Alzheimer’s disease research, to gain new knowledge and become an expert in where this emerging scientific field is moving. In addition, it is not only about receiving a good overview, but also learning to write and defend a successful application for securing funding for Alzheimer’s disease research projects.

## Background

BrightFocus Foundation hosts the Alzheimer’s Fast Track workshop to support early-career researchers who are just entering the Alzheimer’s field. The three-day workshop immerses participants in the current state of the science, provides hands-on training in grant writing, and coaches attendees to perfect presentation skills that are necessary to obtain funding. In addition, it stimulates development of a broader world-wide network of colleagues to advance dementia research. On 16–18 October 2019, the organizers invited experts in science and educational skills necessary for a successful scientific career to present at the Hilton Chicago/Oak Brook Hills Resort and Conference Center outside of Chicago, IL as an official satellite event to the 49th Society for Neuroscience annual meeting.

The BrightFocus funded Alzheimer’s Fast Track (AFT) has been training scientists from around the world for nearly two decades, including its past reincarnation as the “Emerging Concepts in Alzheimer’s Disease” Workshop (for more information on the 2013 workshop, see [9]). Participants have traveled from more than 20 different countries, and are in various stages of their career, ranging from undergraduate student, graduate student, and postdoctoral fellow, through assistant professor to full professor. They come to the AFT to accelerate their knowledge at the outset of their career, to update and hone their current scientific skills, or to make a transition from a scientific focus outside of the sphere of Alzheimer’s disease and related dementia research. For every workshop, the co-chairs update the topics, speakers, and configuration of the workshop to address the newly emerging areas related to Alzheimer’s disease in the global scientific community.

This year, the presentations by invited expert speakers were split into five sessions, with accompanying panel discussion, giving all attendees an opportunity to ask detailed questions and tap into the collective expertise in the room. In addition to the five main sessions, the Alzheimer’s Disease Fast Track workshop included an Elevator Pitch Slam, where each attendee had 1 min to deliver a succinct summary of their research goals and interests, and “Mock Grant Proposal” working groups, where attendees were given time over the course of the 3 days to create a grant proposal from scratch. They verbally have presented and defended as a group in front of their peers and the panel of expert speakers (acting as ‘reviewers’), from whom they were provided real-time feedback on the basics of putting together a successful grant application. This feedback session, chaired by Harry W. M. Steinbusch, PhD (Maastricht University, Netherlands), was unique to each participant and beyond the scope of this meeting summary.

## Meeting

### State of the science: medical; chaired by Cynthia A. Lemere, PhD

Alzheimer’s disease is a neurodegenerative disorder of progressive dementia. During this session, Julie Schneider, MD (Rush University) provided an overview of the disorder’s four key pathways (amyloid/tau, vascular, Lewy body and TDP-43), the relationship to other diseases [1, 3] and factors that contribute to resilience. Craig E.L. Stark, PhD (University of California, Irvine) emphasized the importance of specific MRI-related methods (such as neurite orientation dispersion and density imaging and MR spectroscopy), particularly when correlated with behavior, in the diagnosis and differentiation of Alzheimer’s disease from other age-related pathologies for validating results across species. The complexity of vascular contributions to cognitive impairment and dementia were elaborated in detail by Donna Wilcock, PhD (University of Kentucky) with a detailed overview of the relevant animal models currently in use. Dr. Wilcock also provided an overview of the NIH’s MarkVCID consortium and their ongoing efforts to address these comorbidities in dementia.

A landmark randomized controlled trial for dementia and promotion of healthy aging, the Finnish Geriatric Intervention Study to Prevent Cognitive Impairment and Disability (widely known as the FINGER study, ClinicalTrials.gov Identifier: NCT01041989) investigated the effects of a 2-year multidomain intervention targeting several lifestyle, metabolic and vascular risk factors simultaneously [4, 7]. This is the first intervention with demonstrated cognitive benefits in older adults at increased risk of dementia. This success has been mimicked and leveraged for similar multidomain intervention trials around the world, through the World-Wide FINGERS Network. Francesca Mangialasche, MD, PhD (Karolinska Institutet, Sweden) summarized the findings of the original study and provided perspective on the future of World-Wide FINGERS related projects.

### Professional skills for scientists; chaired by Frank LaFerla, PhD

Although a successful career in science demands knowledge and expertise in the fundamentals of research, the ability to present ideas for funding and results to a variety of audiences is also essential. These so-called ‘soft skills’ are critical differentiators for early-career researchers competing for tenure-track positions. Cindy Lemere, PhD (Brigham & Women’s Hospital) led the session with the basics of applications for the main funder of science – governments. Dr. Lemere gave advice on writing grants that can be applied to any funding source based on her years of experience on both sides of the application process. Rod Corriveau, PhD (National Institute of Neurological Disorders and Stroke) followed with a summary of the NINDS funding strategies and current opportunities for applying for funding for NINDS/NIA projects focusing on Alzheimer’s disease and related dementia, including Lewy Body Dementias, Vascular Contributions to Cognitive Impairment and Dementia (VCID), and Parkinson’s disease dementia. Afterwards, Frank LaFerla, PhD discussed the pitfalls faced by early career academics that he has seen as Dean of the School of Biological Sciences at the University of California, Irvine. Some of his tips for success included knowing the culture of an institution before joining it, telling a story in written and verbal communications for greater impact, teaching and service does matter, keeping a detailed dossier from day 1, focusing your efforts on projects with high research impact, and networking with people in person and on social media. Diane Bovenkamp, PhD (BrightFocus Foundation) summarized some of the current thoughts on work-life balance, emphasizing that skills like communication, time management, finding the right mentors, and taking advantage of all benefits offered by your institution, may help you to achieve your own definition of ‘success’ both within and outside of the laboratory. Ms. Bri McWorter from Activate to Captivate wrapped up the session with an energetic presentation on how to make an impact with elevator pitches and longer powerpoint presentations by carefully controlling one’s voice, word choice, body language and other intangibles. Those skills were immediately reinforced with on-site exercises, to prepare attendees for the individual and group presentations they would make over the course of the workshop.

### The immune system and dementia; chaired by Cynthia A. Lemere, PhD

One critical aspect of neurodegenerative disease is the brain’s immune system reacting to proteinopathies and damaged cells. Microglia and invasive peripheral monocytes become unable to clear plaques from the extracellular space, but are still reacting to their presence. Elizabeth Bradshaw, PhD (Columbia University) discussed how big data approaches are being translated to address the complexities of microglia phenotypes based on one’s genetic background using the example of the CD33 genetic risk allele’s mechanism of action on CD33 and its binding partners to identify novel therapeutic targets in dementia. Katerina Akassoglou, PhD (Gladstone Institute) went into detail on the mechanism for the blood-borne protein fibrinogen triggering microglia activation, which leads to spine loss and aggravates cognitive decline in neurodegenerative diseases [6, 8]. Robert Vassar, PhD (Northwestern University) discussed the promise and challenge of BACE1 inhibition as a therapeutic approach for Alzheimer’s disease. Harry WM Steinbusch, PhD (Maastricht University, Netherlands) wrapped up the session with an overview how epigenetic mechanisms add a layer of complexity in AD and the role of the brainstem in the later stages of aging.

### Understanding and addressing proteinopathies; chaired by Frank LaFerla, PhD

Claudio Cuello, MD, DSc (McGill University, Canada) started off the session with a history of research on Beta-Amyloid and Tau and described the clinical and preclinical deregulation of the brain’s NGF metabolic pathway in the Alzheimer’s pathology. Luc Buée, PhD (Université de Lille, France) followed with an in-depth presentation on the many different roles of tau in the brain and therapeutic strategies [2]. The perspective of the session changed with a presentation by Subhojit Roy, MD, PhD (University of California, San Diego) on the recent advances from his group, using CRISPR/Cas technology to target and manipulate Alzheimer’s-related proteins. Bruce T. Lamb, PhD (Indiana University) wrapped up the session with an update on the MODEL-AD project to develop better rodent models for use in Alzheimer’s disease research.

### Alternative perspectives for Alzheimer’s; chaired by Harry Steinbusch, PhD

WIth the long line of clinical trials for therapeutics developed to clear plaques and tangles failing to meet their primary end points, increasing attention is being paid to new alternatives. William Eimer, PhD (Massachusetts General Hospital) presented an argument for the ‘Antimicrobial Protection Hypothesis’ of Alzheimer’s disease amyloidogenesis. According to this theory, amyloid plaques are an immune reaction to perceived microbial invasion of the brain. The plaques are formed around the pathogen to facilitate clearance but prolonged activation of the pathway results in inflammation and neurodegeneration. Cheil Moon, PhD (Daegu Gyeongbuk Institute of Science and Technology, South Korea) drew attention to the relevance of work on sensory systems, particularly the olfactory sensory (where neurons are directly exposed to the environment) for diagnosing Alzheimer’s disease [10]. This perspective is particularly interesting from a translational aspect since the lack of odor perception is one of the first parameters observed in the early stages of AD. Elizabeth Head, PhD (University of California, Irvine) discussed the research on the connections between Down Syndrome and Alzheimer’s disease [5] with an emphasis on how lessons-learned in each field could benefit the other. She is one of the primary investigators on a BrightFocus funded project on the topic and presented the ongoing results of that project within the context of the literature.

### Big problems in the field; chaired by Frank LaFerla, PhD

Alzheimer’s is much more prevalent among African-Americans, latinos and women while clinical trial participants are predominantly white males. John Morris, MD (Washington University in St. Louis) summarized his work on the racial differences in Alzheimer’s disease which shows that biomarkers and therapeutic strategies need to be adjusted by race. Arguably the most pressing concern for Alzheimer’s therapy development is the difficulty in recruiting human volunteers to clinical studies. The specific challenges facing recruitment, and evidence-based research on how to effectively address the challenges, were covered by Joshua Grill, PhD (University of California, Irvine). Roberta Diaz Brinton, PhD (The University of Arizona Health Sciences), debunked the common myth that the higher prevalence of Alzheimer’s disease among women can be explained by their longer average lifespan by walking through the available data on sex differences in prodromal dementia. Finally, ending the session on an optimistic note, Li-Huei Tsai, PhD (Massachusetts Institute of Technology) presented her work on minimally-invasive stimulation of neurons and microglia within Alzheimer’s patients using transcranial brain wave stimulation.

### Summary

This three-day conference successfully summarized the current state-of-the-science for Alzheimer’s disease research to provide early career and non-dementia researchers with a knowledge base from which to pursue new lines of research. We are optimistic that the impact of this conference will be evident as these scientists go on to develop careers in academia and industry to address the needs of those experiencing dementia and their caregivers. In the near term, the participants left better prepared to create grant proposals and to effectively discuss their research.

There has never been a more exciting time to be researching Alzheimer’s disease and related dementias. Many lessons have been learned from the failures of clinical trials, and the realization that, even though we have known about the existence of Alzheimer’s since it was first described in 1906, we still don’t fully understand how the disease starts and how we can stop it. Hopefully, by convening the best and brightest in the world in workshops such as the BrightFocus Alzheimer’s Fast Track, and by maintaining an unwavering commitment to funding innovative basic, translational and clinical research, we can someday declare that Alzheimer’s and other dementias will no longer devastate our society and families.

## Data Availability

N/A

## Abbreviations

CRISPR: Clustered regularly interspaced short palindromic repeats
FINGER: Finnish Geriatric Intervention Study to Prevent Cognitive Impairment and Disability
MD: Medicinae doctor
MODEL-AD: Model Organism Development and Evaluation for Late-Onset Alzheimer’s Disease
PhD: Philosophiae doctor
